# Synthesis, Molecular Structure Optimization, and Cytotoxicity Assay of a Novel 2-Acetyl-3-amino-5-[(2-oxopropyl)sulfanyl]-4-cyanothiophene

**DOI:** 10.3390/molecules21020214

**Published:** 2016-02-15

**Authors:** Yahia N. Mabkhot, Fahad D. Aldawsari, Salim S. Al-Showiman, Assem Barakat, Saied M. Soliman, Muhammad I. Choudhary, Sammer Yousuf, Taibi Ben Hadda, Mohammad S. Mubarak

**Affiliations:** 1 Department of Chemistry, College of Science, King Saud University, P.O. Box 2455, Riyadh 11451, Saudi Arabia; fzsw2010@hotmail.com (F.D.A.); showiman@ksu.edu.sa (S.S.A.-S.); ambarakat@ksu.edu.sa (A.B.); iqbal.choudhary@iccs.edu (M.I.C.); 2 King Abdulaziz City for Science and Technology, P.O. Box 6086, Riyadh 11442, Saudi Arabia; 3 Department of Chemistry, Rabigh College of Science and Art, King Abdulaziz University, P.O. Box 344, Rabigh 21911, Saudi Arabia; 4 Department of Chemistry, Faculty of Science, Alexandria University, P.O. Box 426, Ibrahimia, 21321 Alexandria, Egypt; saied1soliman@yahoo.com; 5 H.E.J. Research Institute of Chemistry, International Center for Chemical and Biological Sciences, University of Karachi, Karachi-75270, Pakistan; dr.sammer.yousuf@gmail.com; 6 Lab of Chemical Material, Faculty of Sciences, University Mohammed Premier, Oujda 60000, Morocco; taibi.ben.hadda@gmail.com; 7 Department of Chemistry, The University of Jordan, Amman 11942, Jordan

**Keywords:** 2-acetyl-3-amino-5-[(2-oxopropyl)sulfanyl]-4-cyanothiophene, X-ray diffraction, DFT, molecular structure, cytotoxicity

## Abstract

A novel thiophene-containing compound, 2-acetyl-3-amino-5-[(2-oxopropyl)sulfanyl]-4-cyanothiophene (**4**) was synthesized by reaction of malononitrile with CS_2_ in the presence of K_2_CO_3_ under reflux in DMF and the subsequent reaction with chloroacetone followed by cyclization. This compound has been characterized by means of FT-IR, ^1^H-NMR, ^13^C-NMR, and mass spectrometry as well as elemental analysis. In addition, the molecular structures of compound **4** was determined by X-ray crystallography. The geometry of the molecule is stabilized by an intramolecular interaction between N1–H1···O1 to form S6 graf set ring motif. In the crystal, molecules are linked via N1–H2···O1 and C7–H7A···N2 interactions to form a three-dimensional network. Molecular structure and other spectroscopic properties of compound **4** were calculated using DFT B3LYP/6-31G (d,p) method. Results revealed a good agreement between the optimized geometric parameters and the observed X-ray structure. Furthermore, and by employing the natural bond orbital (NBO) method, the intramolecular charge transfer (ICT) interactions along with natural atomic charges at different sites, were calculated; results indicated strong n→π* ICT from LP(1)N5→BD*(2)C15-C16 (63.23 kcal/mol). In addition, the stabilization energy E(2) of the LP(2)O3→ BD*(1)N5-H6 ICT (6.63 kcal/mol) indicated the presence of intramolecular N-H···OH bonding. Similarly, calculations of the electronic spectra of compound **4** using, TD-DFT revealed a good agreement with the experimental data. Finally, compound **4** was evaluated for its in vitro cytotoxic effect against PC-3 and HeLa cell lines, as an anticancer agent, and found to be nontoxic.

## 1. Introduction

Pharmaceutical drugs based on bioactive natural products or small synthetic molecules are still the backbone of cancer therapy, with main cellular targets including tubulin, DNA, and several protein kinases [1,2,3]. Therefore, synthesis of new small organic compounds with selective activity against cancerous cells is the center of attention of anticancer drug development. For example, thiophene privileged structures possess a wide range of biological activities, such as antidepressant [4], analgesic [5], anti-inflammatory [6], anticonvulsant [7,8,9,10], and antimicrobial properties [11]. The currently available active antiepileptic drugs (AEDs), such as brotizolam [12], etizolam [13], and tiagabine [9], contain the thiophene skeleton in their structures. Similarity, sodium phethenylate [9] exhibits high activity due to the presence of a thiophene ring. Furthermore, organic molecules incorporating thiosemicarbazones and hydrazones possess anticonvulsant activity [13,14,15,16].

In view of the wide interest in the activity profile of thiophenes and in the search for new therapeutic agents, and in continuation of our recent work on the synthesis and bioactivity of thiophene derivatives [17,18], we describe herein the synthesis, characterization, X-ray structure determination of a novel thiophene-containing compound, 2-acetyl-3-amino-5-[(2-oxopropyl)sulfanyl]-4-cyanothiophene (**4**). The cytotoxicity of the newly prepared compound was investigated *in vitro* against PC-3 and HeLa cell lines, as a potential anticancer agent. In addition, DFT/B3LYP calculations were performed to study the molecular structural characteristics of the molecule along with its electronic and spectroscopic properties. Similarly, TD-DFT calculations were employed to predict and assign the electronic spectra of the studied compound. Furthermore, NBO calculations were performed to predict the natural atomic charges and to study different intramolecular charge transfer (ICT) interactions in the system.

## 2. Results and Discussion

### 2.1. Synthesis of Compound **4**

**Scheme 1:**
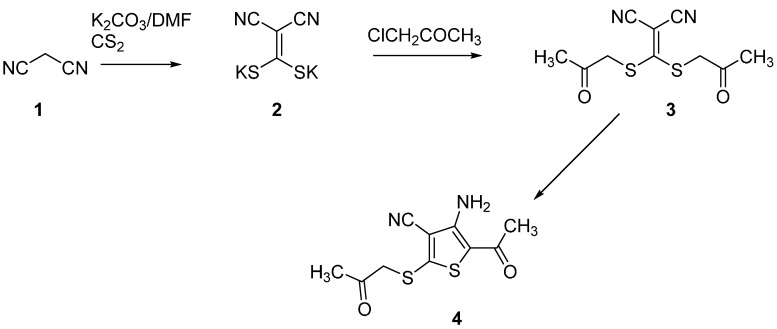
Synthesis of compound **4**.

Compound **4** was synthesized in 78% yield according to the route depicted in Scheme 1. Reaction of malononitrile with CS_2_ in the presence of K_2_CO_3_ under reflux in DMF afforded the intermediate **2**, which upon reaction with chloroacetone afforded the intermediate **3** which cyclized to the new and novel compound **4**. The newly synthesized compound was characterized by elemental analysis and a number of spectroscopic techniques such as IR, MS and NMR. These data, detailed in the experimental section, are consistent with the proposed structure. The mass spectrum of compound **4** exhibited the correct molecular ion peak which is in good agreement with the calculated value. In the ^1^H-NMR spectrum of the prepared compound, we employed DEPT experiments to distinguish between the different types of hydrogens present in the molecule. Finally, single-crystal X-ray diffraction was utilized to confirm the structure of **4**.

### 2.2. Crystal Structure of Compound ***4***

**Figure 1:**
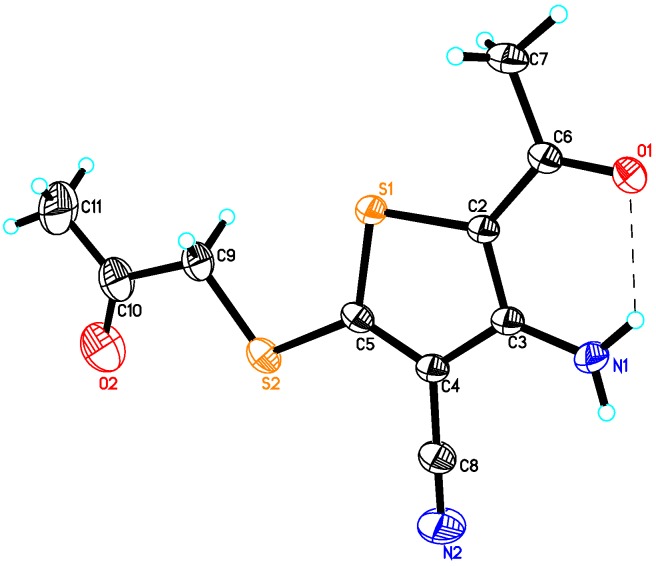
The ORTEP diagram of the final X-ray model of compound **4** with displacement ellipsoids drawn at 30% probability level. H-atoms were placed, and not included in refinement.

The crystal structure of compound **4** is composed of a planar thiophene ring (S1-C2-C3-C4-C5) with an acetyl (O1/C6-C7), a primary amine (N1), (2-oxopropyl)sulfanyl (S2/O2/C9-C11), and a nitrile (N2/C8) substituent, attached to C2, C3, C4, and C5 atoms of planar thiophene ring, respectively, as shown in Figure 1. Crystal and experimental data are listed in Table 1, whereas selected geometrical parameters are given in Table 2.

**Table 1:** The crystal and experimental data of compound **4**.

Empirical formula	C_10_H_10_N_2_O_2_S_2_
Formula weight	254.32
Temperature	293 (2) K
Wave length	0.71073 Å
Crystal system	Orthorhombic
space group	Pmna
Unit cell dimensions	a = 10.6077(7) Å b = 7.0209(5) Å c = 15.9646(10) Å α = β = σ = 90°
Volume	1188.97(14) Å^−3^
Z	4
Calculated density	1.421 mg/m^−3^
Absorption coefficient	0.434 mm^−1^
F(000)	528
Crystal size	0.33 × 0.27 × 0.23 mm
Theta range for data collection	2.31° to 27.50°
Limiting indices	−12 ≤ h ≤ 13, −9 ≤ k ≤ 8, −20 ≤ l ≤ 17
Reflections collected/unique	7832/1477 [R(int) = 0.0266]
Data completeness up to theta 27.50°	100%
Absorption correction	Semi-empirical from equivalents
Max. and min. transmission	0.9068 and 0.8701
Refinement method	Full-matrix least-squares on F^−2^
Data/restraints/parameters	1477/0/0.8701
Goodness-of-fit on F^−2^	1.048
Final R indices [I > 2sigma(I)]	R^1^ = 0.0441, wR^2^ = 0.1151
R indices (all data)	R^1^ = 0.0534, wR^2^ = 0.1248
Largest diff. peak and hole	0.270 and −0.344 e·A^−3^

**Table 2:** Experimental and calculated geometric parameters of compound **4** using DFT B3LYP/6-31G (d,p) method.

Parameter ^a^	Calc.	Exp	Parameter	Calc.	Exp.
R(1-15)	1.769	1.732	A(1-18-2)	125.2	123.9
R(1-18)	1.737	1.702	A(1-18-17)	111.4	112.1
R(2-18)	1.754	1.735	A(18-2-21)	100.6	100.8
R(2-21)	1.831	1.791	A(2-18-17)	123.3	124.0
R(3-19)	1.240	1.224	A(2-21-22)	110.5	109.5
R(4-23)	1.213	1.203	A(2-21-23)	109.0	110.9
R(5-16)	1.346	1.332	A(2-21-26)	110.5	109.5
R(8-20)	1.166	1.122	A(3-19-9)	120.4	120.5
R(9-19)	1.518	1.504	A(3-19-15)	120.4	120.5
R(12-23)	1.515	1.470	A(4-23-12)	122.9	123.4
R(15-16)	1.400	1.411	A(4-23-21)	121.2	120.8
R(15-19)	1.444	1.424	A(5-16-15)	124.1	124.9
R(16-17)	1.444	1.430	A(5-16-17)	123.6	124.5
R(17-18)	1.390	1.375	A(8-20-17)	176.9	179.4
R(17-20)	1.419	1.426	A(9-19-15)	119.1	118.9
R(21-23)	1.527	1.492	A(12-23-21)	115.9	115.8
A(15-1-18)	92.3	92.4	A(16-15-19)	124.8	125.3
A(1-15-16)	110.6	111.3	A(15-16-17)	112.3	110.6
A(1-15-19)	124.6	123.4	A(16-17-18)	113.4	113.6

^a^ Atoms’ numbering according to Figure 3.

**Figure 2:**
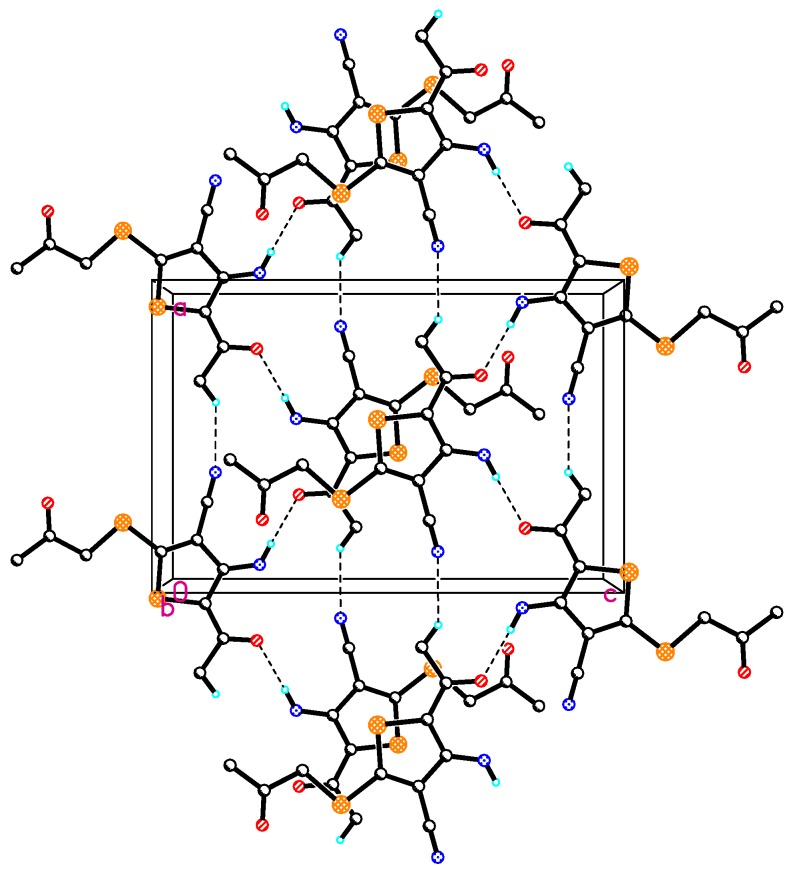
The packing diagram of compound **4** in crystal lattice. Hydrogen atoms not involved in intermolecular hydrogen bonding are omitted for clarity.

The geometry of the molecule is further stabilized by an intramolecular O1–H1···N1 interaction. In the crystal, molecules are linked via N1–H2···O1 and C7–H7A···N2 interactions to form a three dimensional network, as presented in Figure 2 and Table 3.

**Table 3:** Hydrogen bonding data for compound **4**.

D	H	A	D-H	H···A	D···A	D-H···A
N1	H1	O1	0.84(4)	2.13(4)	2.765(4)	132(4)
N1	H2	O1 ^a^	0.89(4)	2.03(4)	2.916(4)	179(3)
C7	H7A	N2 ^b^	0.8600	2.5400	3.359(5)	158.00

Symmetry codes: ^a^ ½ + x, ½ − y, ½ − z, ^b^ −1 + x, −1 + y, −1 + z.

### 2.3. Optimized Molecular Geometry

**Figure 3:**
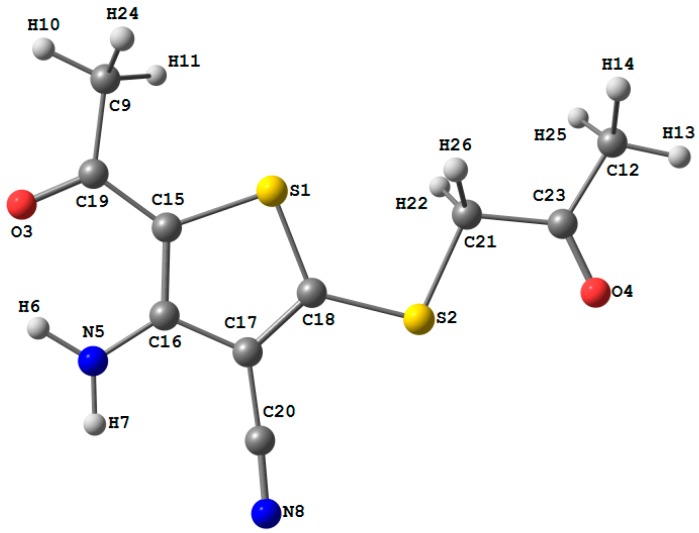
The optimized molecular structure of compound **4**.

Presented in Table 2 are the optimized bond lengths and bond angles for compound **4**, obtained using the B3LYP method with 6-31G (d,p) basis set, whereas the optimized structure is shown in Figure 3; the studied compound possesses a C_1_ point group. In addition, results revealed a good agreement between the calculated geometric parameters (bond distances and bond angles) of compound **4** with those obtained from the crystallographic information file (CIF). In general, most of bond distances are overestimated except for the C15-C16 bond which is slightly underestimated by 0.011Å. Furthermore, the maximum deviations of the calculated bond length and bond angle values from the experimental data are 0.045 Å (C12-C23) and 2.5° (N82-C20-C17), respectively. On the other hand, the calculated intramolecular O---H distance is 1.983 Å (exp. 2.116 Å) indicated the presence of intramolecular H-bonding interaction between the carbonyl O-atom, and one of the amine protons.

### 2.4. Natural Atomic Charge

Atomic charges may have an effect on many properties of molecular systems, such as dipole moment, molecular polarizability, and electronic structure. The calculated natural atomic charges (NAC) at the different atomic sites of compound **4** are presented in Table 4. Results reveal that the O and N atoms of the studied molecule have electronegative natural charges. Of these atomic sites, the N-atom of the amino group was found to be the most electronegative whereas the N-atom of the nitrile group showed the lowest NAC value. In contrast, the S-atoms are electropositive. Furthermore, the natural charge of the ring S-atom is more positive than the one outside the ring. Additionally, all H-atoms are electropositive, where the amino group protons (H6 and H7) are more electropositive than the others. The natural charge values at H6 and H7 are 0.4562 and 0.4287, respectively. Results show that H6 has different natural charge value than H7. The higher NAC value at H6 could be attributed to the intramolecular N-H···OH-bonding interaction.

**Table 4:** The natural atomic charges calculated at the B3LYP/6-31G (d,p).

Atom ^a^	NAC	Atom	NAC
S1	0.4539	H14	0.2587
S2	0.4278	C15	−0.3936
O3	−0.6092	C16	0.2516
O4	−0.5191	C17	−0.2642
N5	−0.8023	C18	−0.3495
H6	0.4562	C19	0.5238
H7	0.4287	C20	0.2624
N8	−0.2969	C21	−0.7120
C9	−0.7706	H22	0.2746
H10	0.2659	C23	0.5892
H11	0.2511	H24	0.2511
C12	−0.7809	H25	0.2587
H13	0.2701	H26	0.2746

^a^ Atoms’ numbering according to Figure 3.

### 2.5. Molecular Electrostatic Potential

**Figure 4:**
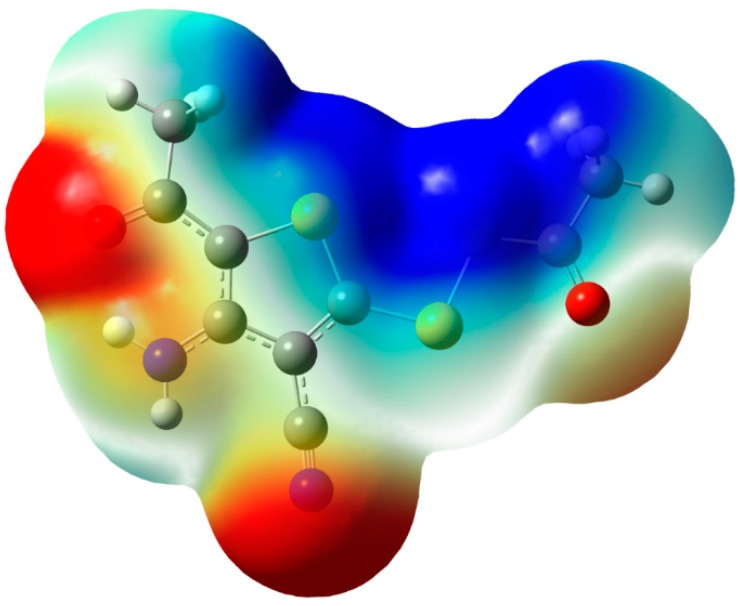
Molecular Electrostatic potentials (MEP) mapped on the electron density surface calculated by the DFT/B3LYP method.

Electrostatic potential maps (MEP) are useful three-dimensional diagrams that can be used to visualize charge distributions and charge related properties of molecules. These maps are used to predict the reactive sites for electrophilic and nucleophilic attacks, and are useful in biological recognition and hydrogen bonding interactions studies [19,20]. In addition, they provide information on the charge distribution and charge related properties of molecules. The MEP of compound **4** calculated, using B3LYP method with 6-31G (d,p) basis set, is shown in Figure 4. Figure reveals that negative regions (red) are mainly localized over the O and N- atoms which are the most reactive sites for an electrophilic attack, whereas the maximum positive regions (blue) are localized over the ring S-atom, and the H-atoms which are the most reactive sites for a nucleophilic attack.

### 2.6. Nonlinear Optical Properties

Nonlinear optical materials were employed as key materials for photonic communications and have been extensively used in industry and medicine [21,22]. Organic compounds with high polarizability (α_0_) and low HOMO-LUMO energy gap (ΔE) are good candidates for nonlinear optical materials. These quantum chemical parameters are obtained from DFT calculations. The α_0_ and ΔE values of compound **4** are calculated to be 165.67 Bohr^3^ and 4.0883 eV, respectively, and its polarizability is 6 times higher than urea. Similarly, the hyperpolarizability (β), is a property related to nonlinear optical properties of molecular systems. The calculated hyperpolarizability (β) values were 901.16 and 69.91 A.U for compound **4** and urea, respectively. Results indicate that compound **4** has about 13 time higher β than urea. In addition, the compound has a lower energy gap (ΔE) compared to urea. Based on these results, compound **4** is considered as a better NLO material than the reference molecule used in the literature [23].

### 2.7. Frontier Molecular Orbitals

**Figure 5:**
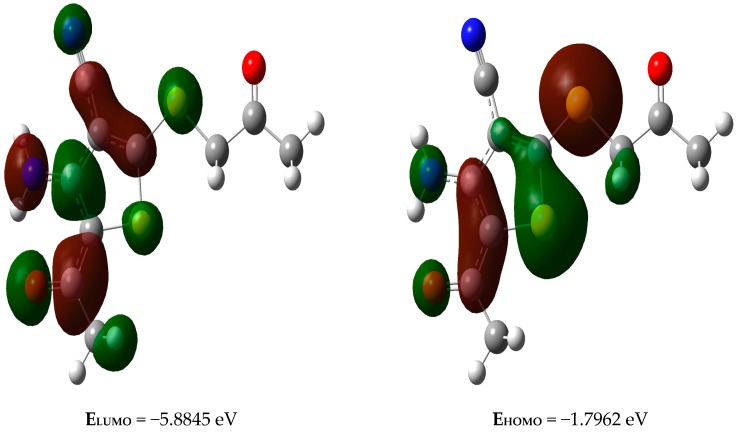
The ground state isodensity surface plots for the frontier molecular orbitals.

Energy and electron densities of the frontier molecular orbitals (FMOs) are very useful for physicists and chemists [24]. Energies of the highest occupied molecular orbital (HOMO) and lowest unoccupied molecular orbital (LUMO) as well as their energy gap reflect the chemical reactivity of the molecule. Moreover, the HOMO-LUMO energy gap has been used to prove the bioactivity from intramolecular charge transfer (ICT) [25,26]. The E_HOMO_ and E_LUMO_ of compound **4** are calculated using the B3LYP/6-31G (d,p) method. The HOMO and LUMO pictures are shown in Figure 5 and the E_HOMO_ and E_LUMO_ are calculated to be −5.8845 eV and −1.7962 eV, respectively. The HOMO-LUMO energy gap (ΔE) represents the lowest energy electronic transition and for compound **4**, the HOMO-LUMO energy gap is 4.0883 eV; this electron transition belongs mainly to π→π* excitations.

**Figure 6:**
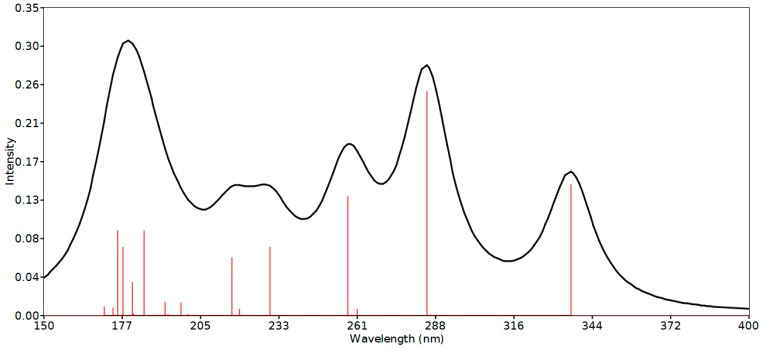
The calculated electronic spectrum of compound **4** using TD-DFT method.

The accurate electronic transitions of the molecule were calculated using the time-dependent density functional theory (TD-DFT). The spin allowed singlet-singlet electronic transitions calculated using the TD-DFT method are listed in Table S1 (Supplementary Material), whereas the calculated electronic spectrum is displayed in Figure 6. Results reveal that compound **4** exhibits five intense electronic transition bands at 336.9, 285.8, 257.9, 230.3, 219.5, and 176.2 nm. The longest wavelength electronic transition band, at 336.9 nm (exp. 374 nm), has a moderate intensity (f = 0.1493) and is assigned to H→L (78%) excitation.

### 2.8. Natural Bond Orbital (NBO) Analysis

Natural bond orbital (NBO) calculations were accomplished to understand the various interactions between the filled NBOs of one bond and the vacant orbitals of another, which is a measure of the intramolecular delocalization of electrons. Presented in Table 5 are stabilization energies E^(2)^, obtained from NBO calculations for the most significant intramolecular charge transfer interactions. The larger the E^(2)^ value, the more intensive the interaction between electron donor and electron acceptor NBOs is, *i.e*. the greater the extent of conjugation of the whole system [27]. The energy of these interactions could be estimated by the second-order perturbation theory [28]. The ICT interactions formed by electron delocalization from π→π*, n→σ*, and n→π* cause stabilization of the system by 31.02, 22.82, and 63.23 kcal/mol, respectively, which is due to BD (2) C15-C16→BD*(2) O3-C19, LP (2) O4→BD*(1) C21-C23, and LP (1) N5→BD*(2) C15-C16 ICT interactions, respectively. These results indicate the presence of strong electron delocalization from LP (1) N5 to the neighboring C15–C16 π*-NBO. Additionally, NBO calculations predicted π→π* electron delocalization from the nitrile group π-system to the neighboring π*-NBO of the C10–C11 bond. Moreover, the ICT LP (2) O3→ BD*(1) N5-H6 stabilization energy E^(2)^ calculated to be 6.63 kcal/mol is a strong evidence about the presence of an intramolecular N-H···OH-bonding interaction.

**Table 5:** The second order perturbation energies E^(2)^ (kcal/mol) of the most important charge transfer interactions (donor-acceptor) of compound **4 ^a^** using B3LYP method.

Donor NBO (i)	Acceptor NBO (j)	E^(2)^ kcal/mol
BD(2)C15-C16	BD*(2)O3-C19	31.02
BD(2)C15-C16	BD*(2)C17-C18	11.99
BD(2)C17-C18	BD*(3)N8-C20	18.60
BD(2)C17-C18	BD*(2)C15-C16	18.69
BD(3)N8-C20	BD*(2)C17-C18	9.01
LP(2)S1	BD*(2)C15-C16	13.30
LP(2)S1	BD*(2)C17-C18	24.70
LP(2)S2	BD*(2)C17-C18	23.97
LP(2)O3	BD*(1)N5-H6	6.63
LP(2)O3	BD*(1)C 9-C19	19.04
LP(2)O3	BD*(1)C15-C19	15.68
LP(2)O4	BD*(1)C12-C23	20.65
LP(2)O4	BD*(1)C21-C23	22.82
LP(1)N5	BD*(2)C15-C16	63.23
LP(1)N8	BD*(1)C17-C20	12.82

^a^ Atoms’ numbering according to Figure 3.

### 2.9. NMR Spectra

**Figure 7:**
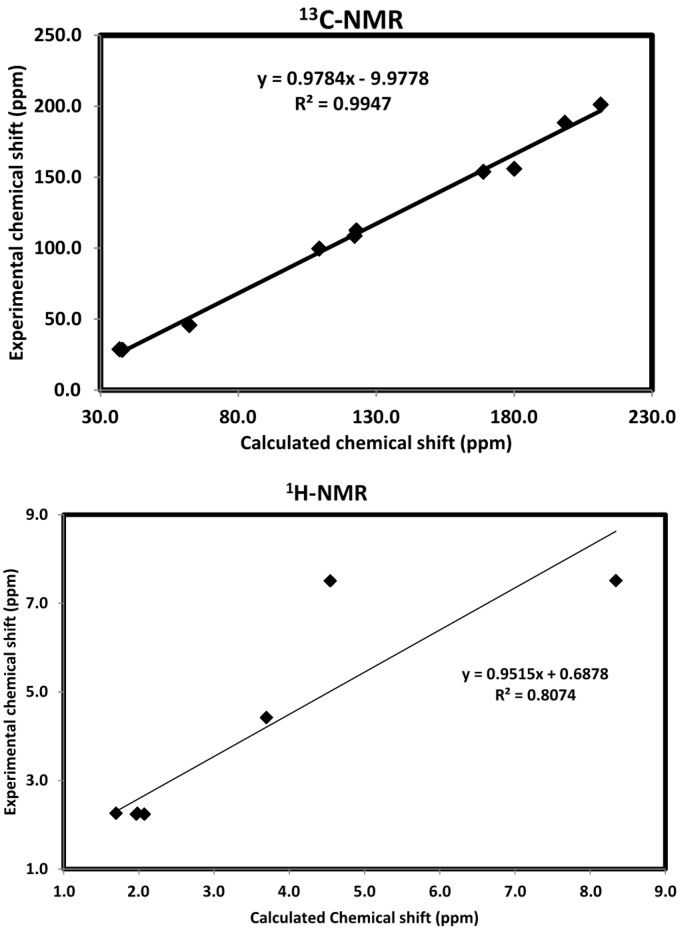
The correlation graphs between calculated and experimental ^1^H- and ^13^C-NMR chemical shifts of compound **4**.

The isotropic magnetic shielding (IMS) values calculated using the GIAO approach at the 6-31G (d,p) level were used to predict the ^13^C- and ^1^H-NMR chemical shifts (δ_calc_) for compound **4**; results were in agreement with the experimental NMR data (δ_exp_) in CDCl_3_. The experimental and theoretical ^1^H- and ^13^C-NMR chemical shifts of the studied compound are presented in Table S2 (Supplementary Material). According to the results, the calculated chemical shifts were in compliance with the experimental findings. As shown in Figure 7, the agreement between experimental and calculated chemical shifts is better for ^13^C (R^2^ = 0.994) than ^1^H (R^2^ = 0.807) [29]. Protons are most likely more affected by the solute intermolecular (solute–solvent) interactions than carbons [22]. Moreover, the presence of N-H···O interaction makes the chemical shift of the H6 strongly deviated from the experimental data. Such intramolecular interaction has no importance in solution where the intermolecular solute-solvent interactions are dominant. If the chemical shift of this proton is omitted from the correlation, a better correlation coefficient will be obtained (R^2^ = 0.917).

### 2.10. Biological Activity Evaluation

Compound **4** was evaluated for its *in vitro* cytotoxic activity against PC-3 and HeLa cell lines; results are presented in Table 6. Results reveal that compound **4** is non-cytotoxic against PC-3 and HeLa cell lines, as a potential anticancer agent, when tested against standard drugs doxorubicin (IC_50_ = 0.912 ± 0.12 μM) and soxorubicin (IC_50_ = 0.306 ± 0.155 μM) as tested standards, respectively, and showed >30% inhibition of PC-3 and HeLa cancer cell lines. This would suggest that compound **4** acts by intercalation of DNA and disruption of topoisomerase II. Furthermore, results also reveal that compound **4** is much less potent than reference drugs. This could be attributed to structural reasons; perhaps the investigated compound is a less efficient intercalating agent than the reference drugs.

**Table 6:** Results of cytotoxicity assays of compound **4**.

Compound	Cytotoxic Activity (PC-3 Cell line) IC_50_ ± SEM [μM]	Cytotoxic Activity (Hella Cell line) IC_50_ ± SEM [μM]
**4**	>30	>30
Standard	Doxorubicin 0.912 ± 0.12	Soxorubicin 0.306 ± 0.155

## 3. Materials and Methods

### 3.1. General Information

Chemicals used throughout this work were purchased from various suppliers, including Sigma-Aldrich (Milwaukee, WI, USA) and Fluka (St. Louis, MO, USA) and were used without further purification, unless otherwise stated. Melting points were measured on a Gallenkamp melting point apparatus in open glass capillaries and are uncorrected. IR spectrum was recorded as KBr pellets on a Nicolet 6700 FT-IR spectrophotometer (Madison, WI, USA). NMR spectra were obtained with the aid of a Bruker Avance AV-600 NMR spectrometer (Hamburg, Germany). ^1^H- (600 MHz) and ^13^C-NMR (150 MHz) were obtained in deuterated dimethyl sulfoxide (DMSO-*d*_6_). Chemical shifts are expressed in δ units whereas coupling constant (*J*) values are given in Hertz (Hz). Mass spectra were acquired with a Jeol, JMS-600 H instrument (Tokyo, Japan). Elemental analysis was carried out on an Perkin Elmer 2400 Elemental Analyzer (Akron, OH, USA), CHN mode and results agreed with the calculated percentages to within the experimental error (±0.4%). Single-crystal X-ray diffraction measurements were performed using a Bruker SMART APEX II CCD diffractometer (Karlsruhe, Germany). In acetonitrile, the electronic absorption spectrum of compound **4** was measured with a Perkin Elmer, Lambda 35, UV/Vis spectrophotometer (Northbrook, IL, USA), and exhibited bands at 262, 289, 365, and 374 nm.

### 3.2. Preparation of 2-Acetyl-3-amino-5-[(2-oxopropyl)sulfanyl]-4-cyanothiophene (**4**)

Compound **4** was prepared according to the following procedure: a mixture of malononitrile (**1**) (0.066 g, 1 mmol) and anhydrous potassium carbonate (10 g) in DMF (30 mL) was stirred vigorously at room temperature for 5 min and then carbon disulfide (0.076 g, 1 mmol) was added with continuous stirring for 30 min. The resulting reaction mixture was cooled in an ice bath, and chloroacetone (0.16 g, 2 mmol) was added with stirring for 15 min. The cooling bath was subsequently removed and the mixture was stirred for 5 h. The solid product **4**, precipitated with the addition of dil. HCl, was collected by filtration, washed with water, and dried. Compound **4** was recrystallized from MeOH to afford colorless crystals. Yield: 78%; m.p. 299–301 °C; IR (KBr, cm^−1^) ν_max_ = 1637 (C=O) cm^−1^; ^1^H-NMR (600 MHz, DMSO-*d*_6_): δ 7.51 (brs, 2H, NH_2_), 4.42 (s, 2H, CH_2_), 2.26 (s, 3H, CH_3_), 2.24 (s, 3H, CH_3_); ^13^C-NMR (150 MHz, DMSO-*d*_6_): δ 201.2, 188.4, 156.0, 153.9, 142.8, 112.6, 108.7, 99.6, 45.8, 28.7, 28.4; DEPT-135 NMR (600 MHz, DMSO-*d*_6_): δ 45.8, 28.7, 28.4; MS *m*/*z* (%): 254 [M+, 100%]; anal. calcd. for C_10_H_10_N_2_O_2_S_2_: C, 47.23; H, 3.96; N, 11.01; S, 25.22; found: C, 47.03; H, 3.88; N, 11.09; S, 25.12.

### 3.3. Crystal Structure Determination

Slow evaporation of a methanol solution of pure compound **4** yielded colorless crystals. A crystal of dimensions 0.33 × 0.27 × 0.23 mm was selected for X-ray diffraction analysis. Data were collected on a Bruker APEX-II diffractometer, equipped with CCD detector and graphite monochromatic MoKα radiation, (τ = 0 71073 A) at 293 (2) K. Cell refinement and data reduction were performed with Bruker SAINT whereas crystal structure was solved with the aid of a SHELXS-97 program [30,31] (Table 1). The final refinement was carried out by full-matrix least-squares techniques with anisotropic thermal data for non-hydrogen atoms on F2. All hydrogen atoms were placed in calculated positions. The crystal structure **4** (Figure 1) was finally refined with R factor of 5.06% for 1477 unique reflections. Molecules were found to be packed in crystal lattice through intermolecular hydrogen bonding (Figure 3, Table 3).

CCDC 1041341 contains the supplementary crystallographic data for this paper. These data can be obtained free of charge via http://www.ccdc.cam.ac.uk/conts/retrieving.html (or from the CCDC, 12 Union Road, Cambridge CB2 1EZ, UK; Fax: +44 1223 336033; E-mail: deposit@ccdc.cam.ac.uk)". This text may be included in the experimental section or as a suitably referenced endnote.

### 3.4. Computational Details

All quantum chemical calculations pertaining to compound **4** were performed by applying DFT method, with the B3LYP functional and 6-31G (d,p) basis set using Gaussian 03 software [32]. The input file was taken from the CIF obtained from the current single crystal X-ray measurement. The geometry was optimized by minimizing energies with respect to all geometrical parameters without imposing any molecular symmetry constraints. GaussView4.1 [33] and Chemcraft [34] programs were employed to draw the structure of the optimized geometry, and to study the frontier molecular orbitals. The molecular electrostatic potential (MEP) was drawn with the aid of a GaussView4.1 program (Semichem Inc., Wallingford, CT, USA) at the B3LYP/6-31G (d,p) optimized structure. Frequency calculations showed the absence of any imaginary frequency modes which confirmed that the optimized structure is an energy minimum. Additionally, the electronic spectra of the studied compound were calculated by the TD-DFT method, whereas the gauge including atomic orbital (GIAO) method was used for the NMR calculations. ^1^H- and ^13^C-NMR isotropic shielding tensors, referenced to the TMS calculations, were carried out at the same level of theory. The natural bond orbital analyses were performed using the NBO calculations as implemented in the Gaussian 03 package at the DFT/B3LYP level [35].

### 3.5. Cytotoxicity Activity by Using MTT Assay

Cytotoxicity activity of compound **4** was evaluated in 96-well flat-bottomed microplates by using the standard MTT (3-[4,5-dimethylthiazole-2-yl]-2,5-diphenyl-tetrazolium bromide, MP) colorimetric assay. For this purpose, the human prostate cancer cell line, PC3, and human cervical cancer cells, HeLa were cultured in Dulbecco’s Modified Eagle Medium, supplemented with 10% of fetal bovine serum (FBS, PAA), 100 IU/mL of penicillin and 100 μg/mL of streptomycin in 75 cm^2^ flasks, and kept in 5% CO_2_ incubator at 37 °C. Exponentially growing cells were harvested, counted with hemocytometer, and diluted with a particular medium with 5% FBS. Cell culture with the concentration of 1 × 10^5^ cells/mL was prepared and introduced (100 μL/well) into 96-well plates. After overnight incubation, the medium was removed and 200 μL of fresh medium was added with different concentrations of compounds (1–30 μM). Stock solution, 20 mM of compounds were prepared in 100% DMSO and final concentration of DMSO at 30 μM is 0.15%. After 48 h, 200 μL MTT (0.5 mg/mL) was added to each well and incubated further for 4 h. Subsequently, 100 μL of DMSO was added to each well. The extent of MTT reduction to formazan within cells was calculated by measuring the absorbance at 570 nm using a micro plate reader (Spectra Max plus, Molecular Devices, CA, USA). Cytotoxicity was recorded as the concentration causing 50% growth inhibition (IC_50_) for PC_3_ and HeLa cancer cells. The percent inhibition was calculated by using the following formula:

(1) % Inhibition = 100 − ((mean of O.D. of test compound − mean of O.D. of negative control)/(mean of O.D of positive control − mean of O.D. of negative control) × 100)

Results (% inhibition) were processed by using Soft-Max Pro software (Molecular Devices, Sunnyvale, CA, USA).

## 4. Conclusions

The synthesis and characterization of the novel compound 2-acetyl-3-amino-5-[(2-oxopropyl)sulfanyl]-4-cyanothiophene (**4**) was successfully achieved in high yield. Structure of the newly synthesized compound was confirmed by single-crystal X-ray diffraction analysis, in addition to various spectroscopic techniques and by elemental analysis. The molecular structure of the studied compound has been optimized using the DFT/B3LYP method and 6-311G (d,p) basis set; calculated bond distances and bond angles showed good agreement with our reported X-ray crystal structure. The molecular electrostatic potential picture of the studied compound has been calculated using the same level of theory. The α_0_ and HOMO-LUMO energy gap (ΔE) values indicated that compound **4** is a better NLO material than urea. Finally, compound **4** was found to be non-toxic against PC-3 and HeLa cell lines, however it is less potent than reference drugs.

## Supplementary Materials

Supplementary materials can be accessed at: http://www.mdpi.com/1420-3049/21/2/214/s1.
