# Supplementary material for: Synthesis, Molecular Structure Optimization, and Cytotoxicity Assay of a Novel 2-Acetyl-3-amino-5-[(2-oxopropyl)sulfanyl]-4-cyanothiophene
Source: Molecules. 2016 Feb 15;21(2):214. doi: 10.3390/molecules21020214 (PMC6274473; doi:10.3390/molecules21020214)
Supplement: Supplementary file 1 [file molecules-21-00214-s001.pdf]

Supplementary Materials: Synthesis, Molecular Structure Optimization, and Cytotoxicity Assay of a Novel 2-Acetyl-3-amino-5-[(2-oxopropyl)sulfanyl]-4-cyanothiophene

Yahia N. Mabkhot, Fahad D. Aldawsari, Salim S. Al-Showiman, Assem Barakat, Saied M. Soliman, Muhammad I. Choudhary, Sammer Yousuf, Taibi Ben Hadda, and Mohammad S. Mubarak

Table S1. Calculated electronic transition bands of compound **4** using TD-DFT method.

λ_{max} (nm)	f	Major contributions
**336.9**	**0.1493**	**H→L (78%)**
309.7	0.0002	H-2→L (84%), H-2→L + 3 (11%)
301.9	0.0002	H→L + 1 (94%)
288.0	0.0000	H→L + 2 (94%)
**285.8**	**0.2552**	**H-1→L (70%), H→L + 3 (11%)**
274.0	0.0001	H-3→L + 1 (92%)
261.2	0.0077	H-1→L + 1 (94%)
**257.9**	**0.1364**	**H→L + 3 (71%)**
254.6	0.0000	H-1→L + 2 (84%), H→L + 4 (12%)
243.6	0.0003	H-1→L + 2 (12%), H→L + 4 (79%)
236.2	0.0000	H-3→L (55%), H-2→L + 3 (36%)
233.9	0.0000	H-3→L (44%), H-2→L + 3 (47%)
230.5	0.0000	H-2→L + 1 (98%)
**230.3**	**0.0781**	**H-4→L (14%), H-1→L + 3 (75%)**
**219.5**	**0.0077**	**H-4→L (20%), H-2→L + 2 (72%)**
216.7	0.0660	H-4→L (56%), H-2→L + 2 (23%), H-1→L + 3 (10%)
216.1	0.0000	H-1→L + 4 (88%)
209.1	0.0004	H→L + 5 (94%)
201.4	0.0009	H-4→L + 1 (98%)
201.2	0.0017	H→L + 6 (79%)
198.7	0.0147	H-3→L + 2 (93%)
197.4	0.0000	H-1→L + 5 (67%)
196.2	0.0000	H→L + 7 (75%)
194.2	0.0014	H-5→L (12%), H-4→L + 2 (62%)
193.8	0.0001	H-5→L (69%), H-1→L + 5 (15%)
193.1	0.0155	H-2→L + 4 (87%)
188.8	0.0009	H→L + 8 (89%)
188.2	0.0000	H-3→L + 3 (99%)
185.7	0.0971	H-4→L + 3 (80%)
181.9	0.0020	H-1→L + 6 (73%), H→L + 10 (17%)
181.5	0.0383	H-6→L (47%), H→L + 9 (30%)
181.5	0.0007	H-8→L (32%), H-1→L + 7 (30%), H→L + 10 (25%)
180.0	0.0005	H-8→L (59%), H→L + 10 (18%)
178.2	0.0784	H-3→L + 4 (87%)
177.2	0.0000	H-1→L + 6 (11%), H-1→L + 7 (51%), H→L + 10 (29%)
176.2	0.0971	H-6→L (22%), H→L + 9 (46%)
175.0	0.0006	H-4→L + 4 (43%), H→L + 12 (20%), H→L + 13 (21%)
174.7	0.0091	H-7→L (78%)
172.4	0.0000	H-4→L + 4 (42%), H→L + 12 (17%), H→L + 13 (30%)
171.5	0.0102	H-2→L + 5 (89%)

Table S2. Calculated chemical shifts δ (ppm) of the compound **4** using GIAO method.

Atom	δ_{calc} (ppm)	$\delta_{exp.}$(ppm)	Atom	δ_{calc} (ppm)	$\delta_{exp.}$(ppm)
H6	8.34	7.51	C9	36.88	28.7
H7	4.54	7.51	C12	37.89	28.4
H10	1.70	2.26	C15	122.19	108.7
H11	1.98	2.26	C16	168.84	153.9
H13	1.97	2.24	C17	109.32	99.6
H14	2.07	2.24	C18	180.06	156
H22	3.70	4.42	C19	198.40	188.4
H24	1.98	2.26	C20	122.83	112.6
H25	2.07	2.24	C21	62.23	45.8
H26	3.70	4.42	C23	211.45	201.2
